# Assessment of reproducibility of cancer survival risk predictions across medical centers

**DOI:** 10.1186/1471-2288-13-25

**Published:** 2013-02-20

**Authors:** Hung-Chia Chen, James J Chen

**Affiliations:** 1 Division of Bioinformatics and Biostatistics, National Center for Toxicological Research, U.S. Food and Drug Administration, 72079, Jefferson, AR, USA; 2 Graduate Institute of Biostatistics and Biostatistics Center, China Medical University, Taichung, Taiwan

## Abstract

**Background:**

Two most important considerations in evaluation of survival prediction models are 1) predictability - ability to predict survival risks accurately and 2) reproducibility - ability to generalize to predict samples generated from different studies. We present approaches for assessment of reproducibility of survival risk score predictions across medical centers.

**Methods:**

Reproducibility was evaluated in terms of consistency and transferability. Consistency is the agreement of risk scores predicted between two centers. Transferability from one center to another center is the agreement of the risk scores of the second center predicted by each of the two centers. The transferability can be: 1) model transferability - whether a predictive model developed from one center can be applied to predict the samples generated from other centers and 2) signature transferability - whether signature markers of a predictive model developed from one center can be applied to predict the samples from other centers. We considered eight prediction models, including two clinical models, two gene expression models, and their combinations. Predictive performance of the eight models was evaluated by several common measures. Correlation coefficients between predicted risk scores of different centers were computed to assess reproducibility - consistency and transferability.

**Results:**

Two public datasets, the lung cancer data generated from four medical centers and colon cancer data generated from two medical centers, were analyzed. The risk score estimates for lung cancer patients predicted by three of four centers agree reasonably well. In general, a good prediction model showed better cross-center consistency and transferability. The risk scores for the colon cancer patients from one (Moffitt) medical center that were predicted by the clinical models developed from the another (Vanderbilt) medical center were shown to have excellent model transferability and signature transferability.

**Conclusions:**

This study illustrates an analytical approach to assessing reproducibility of predictive models and signatures. Based on the analyses of the two cancer datasets, we conclude that the models with clinical variables appear to perform reasonable well with high degree of consistency and transferability. There should have more investigations on the reproducibility of prediction models including gene expression data across studies.

## Background

Providing guidance on specific therapies for pathologically distinct tumor types/stages to maximize treatment efficacy and minimize toxicity is an important goal in clinical oncology [1-6]. The development of prediction models using the TNM staging system, primary tumor (T), regional lymph nodes (N), and distant metastasis (M), with using basic clinical covariates to classify target patients as high-risk or low-risk for treatment recommendation has been used for more than a decade.

Recent developments in microarray technology have accelerated research in the development of genomic biomarker classifiers for safety assessment, disease diagnosis and prognosis, and prediction of response for patient assignment [6-10]. Several microarray studies have shown an association between patient survival and gene expression profiles [10-20]. Some recent publications have investigated the use of microarray gene expression data alone or in combination with the clinical covariate variables [21-23] as an improvement over the standard approach of using only clinical variables in estimating patient survival. It is well known that use of all genes to develop a microarray-based prediction model can suppress its performance. Selection of a subset of relevant genes to enhance predictive performance becomes an important part in developing a microarray-based classifier. However, at the present time, there is no consensus about what types of algorithms are best for modeling gene expression data alone or in combination with clinical variables for binary prediction. Selection of the most relevant genes to develop prediction models for survival risk presents additional challenges.

In the evaluation of a prediction model, two most important considerations are 1) predictability (predictive performance) – ability to predict the survival risks of patients accurately and 2) reproducibility (generalizability) - ability of the model to predict new samples generated from different locations or on different times. A good prediction model should perform well in both predictability and reproducibility. A model with a higher reproducibility does not necessarily imply better predictability; it should be noted that reproducibility is valid only when the model has a good predictability. Evaluation of the performance of prediction models for binary outcomes has been well studied for data generated from a single medical center. The performance of a binary classifier is typically evaluated in terms of the positive and negative predictive values, sensitivity and specificity, and/or accuracy in terms of the number of true and false positives, and the number of true and false negatives. In contrast, when the outcome is survival time in the presence of censored observations, the measure of predictability is less apparent. Survival prediction modeling is usually performed to classify patients into two or more risk groups, not to predict exact survival time so that patients would be treated based on the risk group classification. Common measures to assess the predictive performance of a survival prediction model include the hazard ratios, significant difference in the Kaplan-Meier survival cures between identified risk groups, the concordance index [24-26], Brier scores [27], absolute measure of predictive accuracy [28] and several others [29-33]. Together, these measures evaluate different aspects of predictability of the model and its ability to accurately characterize patient’s survival risk.

Assessment of the generalizability of a prediction model is to determine whether its performance is reproducible for similar data generated from either same or different locations and/or different times. A prediction model is to be applied to predicting new samples. In addition that the model should perform well in predicting the samples obtained from the current study, its predictive performance must be generalizable across different studies. A prediction model developed from one study, that has been shown to perform well, might not be reproduced its performance when it is applied to other studies. The issue of the lack of reproducibility of predictive signatures and predictive models across studies has been aware. There were several large-scale screening studies [34-36] have identified several gene signatures with high predictive performances in their original discovery dataset, yet a recent report has indicated that these signatures are seldom in common across different studies [37]. For example, Shedden et al. [21] attempted unsuccessfully to validate the signatures reported by Chen et al. [13]. The lack of reproducibility makes these biomarkers difficult to be applied in clinical usage for treatment recommendation.

Justice et al. [38] considered the two terms for assessing a prognostic system: *accuracy* (calibration and discrimination) and *generalizability* (reproducibility and transportability). They defined *calibration* as “predicted probability is neither too high nor too low” for an individual patient and *discrimination* as “relative ranking of individual risk is in correct order”. *Reproducibility* was defined as “ability to produce accurate predictions among patients not included in the development of the system but from the same population”, and *transportability* as “ability to produce accurate predictions among patients drawn from a different but plausibly related population”. Note that assessment of *accuracy* is to evaluate the predictive performance. However, in the context of Justice et al. [38] the *accuracy* assessment covers both the predicting probabilistic risk of an individual patient (*calibration*) and ranking of his/her risk as compared to other patients (*discrimination*). This paper focuses only on the evaluation of the ranking of risks to match observed survival times and classifying patients into risk categories accordingly. Furthermore, *generalizability* defined by Justice et al. [38] consisted of *reproducibility* (internal validity) and *transportability* (external validity). Predictive performance (or accuracy) should be evaluated based on the patients that are not included in the model development [7,39]. Typically, the current samples are used in two ways: (i) as training samples to develop the prediction model and (ii) as test (future) samples to assess predictive performance [7,39-41]. That is, assessment of *reproducibility* within a study has been integral part of model development. A prediction model developed from a single study which does not reflect many sources of variability outside research conditions such as historical, geographic, methodologic, spectrum, and follow-up interval aspects described in Justice et al. [38], represents an internal validation [39]. In this paper, reproducibility refers the ability to produce performance on patients from other studies, *transportability*. Therefore, “reproducibility” has the meaning as “generalizability”. The term “reproducibility” is a common terminology used in the evaluation of different platforms, studies, gene signatures, etc. [42,43]. More detailed approaches and discussions on the development of a prediction model from a single study are given in the Discussion section. The definitions of the terminologies considered in this paper are summarized in Table 1.

**Table 1:** Definitions of key terms

**Term**	**Definition**
Predictability (Predictive performance)	Ability of a model to predict risk scores of patients that can match their survival risks (not survival times).
Generalizability (Reproducibility)	Ability of a model to predict risk scores of patients generated from different studies (different locations or different times).
Consistency	Agreement between two centers to predict the risk scores of a targeted center.
Transferability.	Agreement between one center and the targeted center to predict risk scores of targeted center.
Internal validation	An assessment of predictive performance of a model in which the available data are divided into a training set and a test set, the model is developed in the training set and applied to the test set.

The primary objective of this paper is to present approaches to investigating reproducibility of predictive models and signatures across different medical centers. Reproducibility across centers is evaluated in terms of consistency and transferability. Consistency is the agreement of risk scores predicted between two centers. Transferability from one center to another center is the agreement of the risk scores of the second center predicted by each of the two centers. We considered eight risk prediction models based on established approaches for modeling clinical variables and microarray gene expression data. Two recent studies on lung cancer [21] and colon cancer [20], where data were collected from more than one center, are used in the evaluation of the predictability and generalizability of predictive models and signatures.

The first step in the evaluation of reproducibility of a prediction model is to assess its predictability. In theory, some models may have a good predictability but a poor reproducibility, or vice versa. Models with high predictability and reproducibility are obviously desirable. Since standard measures to assess predictability of survival prediction models have not been fully established, various predictability measures are considered in the evaluation. The predictability and reproducibility measures of each of the eight models are calculated to assess the overall performance of each model. However, we do not attempt to propose or identify the best approach/model to predict patient survival risk, the purpose is to illustrate the differences among the eight models.

## Methods

### Models developed from training dataset

Eight survival prediction models to estimate patient survival risk were considered. These eight models included two clinical models, two gene expression models, and four models based on combinations of the two clinical and two gene expression models. The two clinical models were 1) the Cox proportional hazards model (Model A) and 2) the regression tree (Model B), these are two well-established methods for modeling survival data. All clinical variables including AJCC (The American Joint Committee on Cancer) stage, gender, age, and histology were considered in both models. The Cox proportional hazards model approach involved fitting the relevant clinical variables to a multivariate Cox model [44]. The regression tree modeling approach consisted of two steps. The first step was to use a standard survival tree model [45-50] to classify patients into different risk groups according to their incidence rates. The second step involved fitting a univariate Cox model using the patients’ incidence rates as an independent variable.

It is well known that gene expression data typically involve a large number of genes; selection of a subset of relevant genes to enhance predictive performance becomes an important part in the model development. The data were first analyzed using the univariate Cox model to select a set of “significant” genes. There still could be too many significant genes in a model, which could make the model estimate unstable. The dimensional reduction using principal component analysis can be applied to extract the *k* relevant meta-genes, the linear combinations of the all selected genes. The *k* can be tuned by cross-validation, but we set *k*=5. An alternative approach is to select the k most significant genes to develop the model, we set k = 10. For the set of selected genes, two gene expression models were developed using a multivariate Cox model with covariates provided by 1) the first five principal components (Model C) and 2) the top 10 ranked genes (Model D). Each gene expression model was additively combined with each clinical model to develop four clinical and gene expression models: E=A+C, F=A+D, G=B+C, and H=B+D. A summary of the eight models is given in Additional file 1: Table S1.

### Assessment of predicted risk scores for the patients in test dataset

The regression coefficients of the fitted Cox model developed from the training data, ${\hat{\beta }}_{\mathrm{train}}$, were used to compute the predictive risk scores for each patient in test data, ${\hat{\beta }}_{\mathrm{train}}^{T}x_{\mathrm{test}}$. The predictive risk scores were then used to compute predictive performance measures to evaluate the survival prediction model built from the training data. Although the continuous risk scores for test data are adequate to rank the risk levels, clinicians often use the stratified risk groups to exhibit the risk categories to the patients. Therefore, both approaches are considered in the evaluation: single-group analysis and two-group comparison.

In the single-group analysis, the p-value of hazard ratio ($e^{\hat{\beta }}$), R^2^, Somers’ rank correlation D_xy_[26], and time dependent receiver operating characteristic (ROC) curve are obtained to evaluate the predictive scores. The p-value of hazard ratio and R^2^ are calculated from the fitted univariate Cox model of the predictive risk scores. The Somers’ rank correlation D_xy_ and R^2^ measure the goodness-of-fit in terms of agreement and explained variation between the risk scores and survival times, respectively. ROC curve is a measure of predictive ability of binary classifiers, and Hegerty et al. [30] firstly applied it to develop the time dependent ROC, ROC(t), curve for censored survival data to evaluate a diagnostic marker. They have shown that it can lead to inconsistence of the negative probability mass if the true positive rates, TPR(t), and false positive rates, FPR(t), for ROC(t) curve are estimated by the conditional probability. However, the ROC(t) curve in this paper does not result in the inconsistence (Additional file 1).

The two-group comparison is the most frequently used approach for performance assessment. The test data are first segregated into high-risk and low-risk groups by a cutoff threshold, and the Cox model or log-rank test is then applied to compare the difference in survival time between the two groups. This approach depends on the choice of threshold. We use the median of the training scores as the threshold. A significant p-value implies that the survival times between the high-risk and low-risk group ranked by the risk scores are different significantly. We calculated both p-values of the hazard ratio in Cox model and log-rank test for completeness.

### Correlation coefficient for measure of consistency and transferability

Reproducibility of survival risk predictions was evaluated in terms of the two measures: consistency and transferability. Both are measures of an agreement of predictive risk scores predicted by two centers. Consistency is the agreement between two centers to predict the risk scores of another center, which can be one of the two centers or an independent third center. Transferability from one center to another center is the agreement of the risk scores of the second center predicted by each of the two centers. The transferability can be in terms of 1) whether a predictive model developed from one center can be applied to predict the survival risk for the patients from other centers (model transferability) or 2) whether signature markers of a predictive model developed from one center can be applied to predict patients from other centers (signature transferability). Both consistency and transferability are measures of an agreement of two centers to predict risk scores of a targeted center. The transferability characterizes the applicability of a model built from one center and applied to the targeted center. Consistency characterizes an agreement between two centers to predict a targeted center. Consistency is a general terminology covering two or more centers. Since the agreement between two centers is of the primary interest, assessment of transferability is more useful.

Agreement between two centers is evaluated using the Pearson’s correlation coefficient.

The transferability from center *i* to center *j* can be expressed mathematically as $\mathrm{Tra}n_{\mathrm{ij}}=\rho \left({\hat{\beta }}_{i}^{T}X_{j},{\hat{\beta }}_{i}^{T}X_{j}\right),$ and the consistency of centers *i* and *j* to predict center *k* can be expressed as $\mathrm{Con}s_{\mathrm{ij}|k}=\rho \left({\hat{\beta }}_{i}X_{k},{\hat{\beta }}_{j}X_{k}\right)$, where *X*_*i*_ and *ρ* are the predictor matrix and the Pearson correlation coefficient and ${\hat{\beta }}_{i}$, $\;{\hat{\beta }}_{j}$ and $\;{\hat{\beta }}_{k}$ are the coefficients of the fitted models developed from the centers *i, j,* and *k*, respectively. When *k = j* (or *i*), the consistency is identical to transferability from center *i* to *j* (or from *j* to *i*). The coefficient ${\hat{\beta }}_{i}$ can be the estimate from the model developed using the entire dataset or using a partial dataset in the center *i*. The use of entire dataset will evaluate the transferability once, i.e., the correlation coefficient between the two sets of predicted scores developed by two centers is computed once. On the other hand, the use of partial data can compute the correlation coefficient multiple times with different sets of partial data. In the analysis of two cancer datasets shown below, ${\hat{\beta }}_{i}$ is estimated based on the entire dataset for the lung cancer data and is estimated based on the partial dataset for the colon data (Results).

## Results

### Lung cancer

The lung cancer dataset was composed of four datasets with a total of 442 patients generated from the Directors’s Challenge Consortium at four medical centers: University of Michigan Cancer Center (UM), Moffitt Cancer Center (HLM), Dana-Farber Cancer Institute (DFCI), and Memorial Sloan-Kettering Cancer Center (MSK) (https://array.nci.nih.gov/caarray/project/jacob-00182) [21]. The four datasets used a common platform for the data collection. Gene expression data were generated by Affymetrix 133A chips and the expression values were calculated using the robust multi-array average (RMA) algorithm [51]. The number of patients from each institution were 177 (UM), 79 (HLM), 82 (DFCI), and 104 (MSK). The clinical covariates included age, gender, lymph nodes N, tumor stage T, and histology; the samples or genes with missing values were excluded from the analysis.

For illustrative purpose, we first followed the analysis of Shedden et al. [21], who utilized the UM and HLM data as the training set and the MSK and DFCI data as the test set. An additional analysis was performed, in which the roles of the training data and test data were interchanged. Estimates of several predictive performance measures from the risk scores predicted by the eight models are given in Tables 2, 3, 4, and 5. Performance measures obtained from the two analyses are consistent, except for the case when Model C is used to test the UM and HLM datasets (Tables 4 and 5). Model C has small estimates of absolute D_xy_, HR, and R^2^, yet shows significance in the single-group analysis (p=0.021) and non-significant difference in the two-group comparison (p=0.132 and 0.129 for Cox model and log-rank test).

**Table 2:** Performance evaluation using single-group analysis for the lung cancer data (training data: UM and HLM; test data: MSK and DFCI)

**Model**	**D**_**xy**_	**HR**	**P-value**	**R**^**2**^
A	−0.420	3.34	1.50E-8	0.169
B	−0.244	1.59	2.19E-4	0.059
C	−0.093	1.04	0.629	0.001
D	−0.050	1.05	0.655	0.001
E	−0.196	1.19	0.031	0.026
F	−0.265	1.51	8.46E-4	0.062
G	−0.170	1.15	0.083	0.017
H	−0.333	1.71	2.50E-5	0.083

Columns 3–5 are the estimates from fitting a Cox model using the predicted risk scores as an independent variable.

**Table 3:** Performance evaluation using two-group comparison for the lung cancer data (training data: UM and HLM; test data: MSK and DFCI)

**Model**	**Cox Model**		**Log-rank Test P-value**
	**HR**	**P-value**	
A	3.78	1.18E-6	1.81E-7
B	2.03	0.003	0.002
C	1.34	0.264	0.261
D	0.94	0.808	0.811
E	1.99	0.006	0.005
F	1.91	0.009	0.007
G	1.57	0.068	0.066
H	2.37	0.005	0.004

The high-risk and low-risk groups for test data were segregated based on the median of the training scores. These are results from fitting a Cox model using the risk group as an independent variable and from the log-rank test.

**Table 4:** Performance evaluation using single-group analysis for the lung cancer data (training data: MSK and DFCI; testing data: UM and HLM)

**Model**	**D**_**xy**_	**HR**	**P-value**	**R**^**2**^
A	−0.303	1.98	9.37E-11	0.149
B	−0.230	1.61	2.18E-9	0.109
C	−0.183	1.31	0.021	0.020
D	−0.119	1.11	0.177	0.007
E	−0.342	1.70	3.61E-11	0.150
F	−0.237	1.30	1.20E-5	0.067
G	−0.269	1.51	4.49E-8	0.101
H	−0.258	1.37	5.19E-7	0.086

**Table 5:** Performance evaluation using two-group comparison for the lung cancer data (training data: MSK and DFCI; testing data: UM and HLM)

**Model**	**Cox Model**		**Log-rank Test P-value**
	**HR**	**P-value**	
A	2.40	6.00E-8	2.37E-8
B	1.74	5E-4	4.21E-4
C	1.53	0.132	0.129
D	1.33	0.138	0.137
E	2.10	3.99E-5	2.66E-5
F	1.64	0.002	0.002
G	1.74	0.002	0.001
H	1.74	6.43E-4	5.48E-4

In Tables 2,3,4 and 5, the clinical models (Models A and B) appear to perform better than the gene expression models (Models C and D). In Tables 2 and 3, Models A and H appear to perform the best; in Tables 4 and 5, Models A and E perform the best. The prediction models with both clinical and gene expression variables (Models E, F, G, and H) show little or no improvement over the clinical models. In summary, Model A appears to perform the best among the eight models. An ROC analysis of the 8 models estimated at month 36 after surgery confirms our findings (Additional file 1: Figure S1), and Model A is also the best one. The predictive abilities of the eight models are also evaluated by the pairwise between center predictions. Each center can be predicted by three other centers for a total of 12 pairwise predictions (Additional file 1: Table S2-S5). Models A, E, and G appear to perform the best among the eight models; Model A performs the most consistently well. These results are in agreement with the above analysis. For Models A, E and G, HLM predicting DFCI has the best performance, it has the best performance in compared with the results from other two center predictions. MSK predicting HLM, DFCI predicting MSK and MSK predicting DFCI have poorest prediction ability for Models A, E, and G, respectively.

The four lung cancer datasets were further evaluated to address the issue of reproducibility of the estimated risk scores among the four medical centers. Each center has its own training scores and three risk scores subsequently predicted by the models developed from the other three centers. For each center, transferability and consistency of the patients’ estimated risk scores across centers were calculated through the use of pairwise correlation analyses. Pairwise correlation estimates resulting from the eight prediction models are given for each medical center in Table 6. Correlation coefficients listed in the first three rows in each panel measure model transferability. The last three rows show pairwise correlation coefficients between the risk scores predicted by any two of the other three centers; the last three rows measure the consistency between two centers to predict a third center. The correlation coefficients in Table 6 are not a measure of signature transferability since the four sets of risk scores were predicted by four different classifiers with different predictors.

**Table 6:** Estimates of correlation between predicted risk scores from a center’s own training model and predicted risk scores using the training model of another center (training center) are given in the first three rows of each table (model transferability)

**Center**	**Training Center**	**A**	**B**	**C**	**D**	**E**	**F**	**G**	**H**
UM	HLM	0.74	0.62	0.56	0.33	0.58	0.43	0.56	0.42
	DFCI	0.77	0.56	0.56	0.14	0.71	0.32	0.65	0.27
	MSK	0.66	0.47	0.41	0.25	0.49	0.34	0.47	0.31
	HLM and DFCI	0.83	0.78	0.54	0.19	0.46	0.4	0.59	0.39
	HLM and MSK	0.58	0.38	0.39	0.22	0.34	0.23	0.33	0.18
	DFCI and MSK	0.5	0.36	0.2	0	0.3	0.11	0.35	0.15
HLM	UM	0.65	0.63	0.49	0.09	0.63	0.25	0.53	0.27
	DFCI	0.83	0.77	0.5	0.21	0.53	0.34	0.64	0.37
	MSK	0.53	0.18	0.27	0.37	0.38	0.4	0.28	0.33
	UM and DFCI	0.68	0.66	0.5	0.25	0.75	0.47	0.65	0.47
	UM and MSK	0.72	0.46	0.34	0.12	0.54	0.29	0.54	0.2
	DFCI and MSK	0.44	0.27	0.2	0.03	0.47	0.24	0.49	0.19
DFCI	UM	0.84	0.62	0.6	0.29	0.72	0.54	0.65	0.43
	HLM	0.86	0.87	0.53	0.35	0.56	0.43	0.67	0.48
	MSK	0.56	0.41	0.19	0.34	0.23	0.38	0.31	0.36
	UM and HLM	0.72	0.62	0.52	0.37	0.61	0.55	0.6	0.51
	UM and MSK	0.7	0.54	0.44	0.34	0.51	0.38	0.51	0.28
	HLM and MSK	0.61	0.34	0.41	0.27	0.38	0.28	0.3	0.17
MSK	UM	0.7	0.5	0.35	0.35	0.45	0.46	0.46	0.36
	HLM	0.66	0.19	0.5	0.39	0.45	0.3	0.32	0.29
	DFCI	0.54	0.21	0.41	0.06	0.32	0.11	0.41	0.11
	UM and HLM	0.75	0.55	0.42	0.14	0.55	0.32	0.47	0.27
	UM and DFCI	0.76	0.52	0.7	0.35	0.71	0.42	0.6	0.39
	HLM and DFCI	0.88	0.77	0.59	0.28	0.48	0.52	0.62	0.49

The last three rows (consistency) display the correlation between predicted risk scores of the “center” using the training models developed by the “training centers.”

In general, a good prediction model shows a high cross-center consistency. Model A shows the best cross-center consistency among the eight models. Centers HLM and DFCI show excellent agreement with correlation coefficients for the four centers ranging between 0.83 and 0.88 when each center is used as the training center. UM, HLM, and DFCI appear to have reasonable agreement. MSK shows poor agreement with the other three centers. Additional file 1: Figure S2 shows the pairwise scatter plots for Model A (Additional file 1: Figure S2). Besides, Model D for DFCI and MSK has smallest consistency correlation to predict UM (ρ =0) and HLM (ρ =0.03), and it also has poor transferability from DFCI to MSK (ρ =0.06). Model D from the MSK training model showed poor predictive ability for DFCI (Additional file 1: Table S5c).

### Colon cancer

The colon dataset consisted of 232 patients from two medical centers: 55 patients from Vanderbilt Medical Center (VMC) and 177 patients from Moffitt Cancer Center (MCC). The clinical covariates include age, gender, and AJCC stage (http://ncbi.nlm.nih.gov/geo/query/acc.cgi?acc=GSE17538) [20]. RMA was used to pre-process/normalize gene expression data (Affymetrix 133Plus chips). There were three types of survival data: overall survival, disease specific survival, and disease free survival; however, only the overall survival time variable was evaluated. We follow the analysis of Smith et al. [20] and use data from the 55 VMC patients as the training data and data from the 177 MCC patients as the test dataset. The samples or genes with missing values are not included in the analysis.

Table 7 and 8 show estimates of several performance measures for the eight prediction models from the single-group analysis and two-group comparison. The median value of risk scores in the training data for Models C, E, and G could not generate two risk groups for comparison. For Model E all patients were in one group; for Models C and G the patients in one of two groups were all censored (Additional file 1: Figure S3). The ROC curves of patient survival evaluated at months 23, 42, and 68 after surgery, which corresponds to the 25th, 50th, and 75th percentiles of the follow-up time, respectively, are shown in Additional file 1: Figure S4. Models A, B, and G show the best performance. Note that the c-index=−0.6033 (or equivalently D_xy_=−0.2066) in Smith et al. [20] is much smaller than the D_xy_ values listed for the aforementioned best performing models.

**Table 7:** Performance evaluation of the colon cancer data for eight prediction models

**Model**	**D**_**xy**_	**HR**	**95% C.I.**		**P-value**	**R**^**2**^
A	−0.520	3.85	2.27	6.51	5.04E-7	0.283
B	−0.488	5.13	3.09	8.51	2.46E-10	0.251
C	−0.227	2.48	1.52	4.04	2.78E-4	0.043
D	−0.126	1.33	0.83	2.11	0.233	0.021
E	−0.446	4.18	2.47	7.06	8.99E-08	0.169
F	−0.210	1.87	1.16	3.00	0.01	0.049
G	−0.563	4.52	2.66	7.67	2.25E-08	0.293
H	−0.361	2.29	1.42	3.68	6.5E-4	0.149

Somers’ correlation (D_xy_), the hazard ratio (*HR*) with the 95% confidence limits (*CI*) and p-value of significance (P-value), and the coefficient of determination (R^2^) are given for the single-group analysis.

**Table 8:** Performance evaluation of the colon cancer data for eight prediction models

**Model**	**HR**	**95% C.I.**		**P-value**	**R**^**2**^
A	3.73	2.25	6.16	2.90E-7	0.152
B	5.13	3.09	8.51	2.46E-10	0.220
C	NA	NA	NA	NA	NA
D	1.73	0.93	3.22	0.083	0.019
E	NA	NA	NA	NA	NA
F	2.12	1.21	3.69	0.008	0.044
G	NA	NA	NA	NA	NA
H	2.68	1.23	5.85	0.013	0.044

The hazard ratio (*HR*) with the 95% confidence limits (*CI*) and p-value of significance (P-value), and the coefficient of determination (R^2^) are given for the two-group comparison.

**Figure 1:**
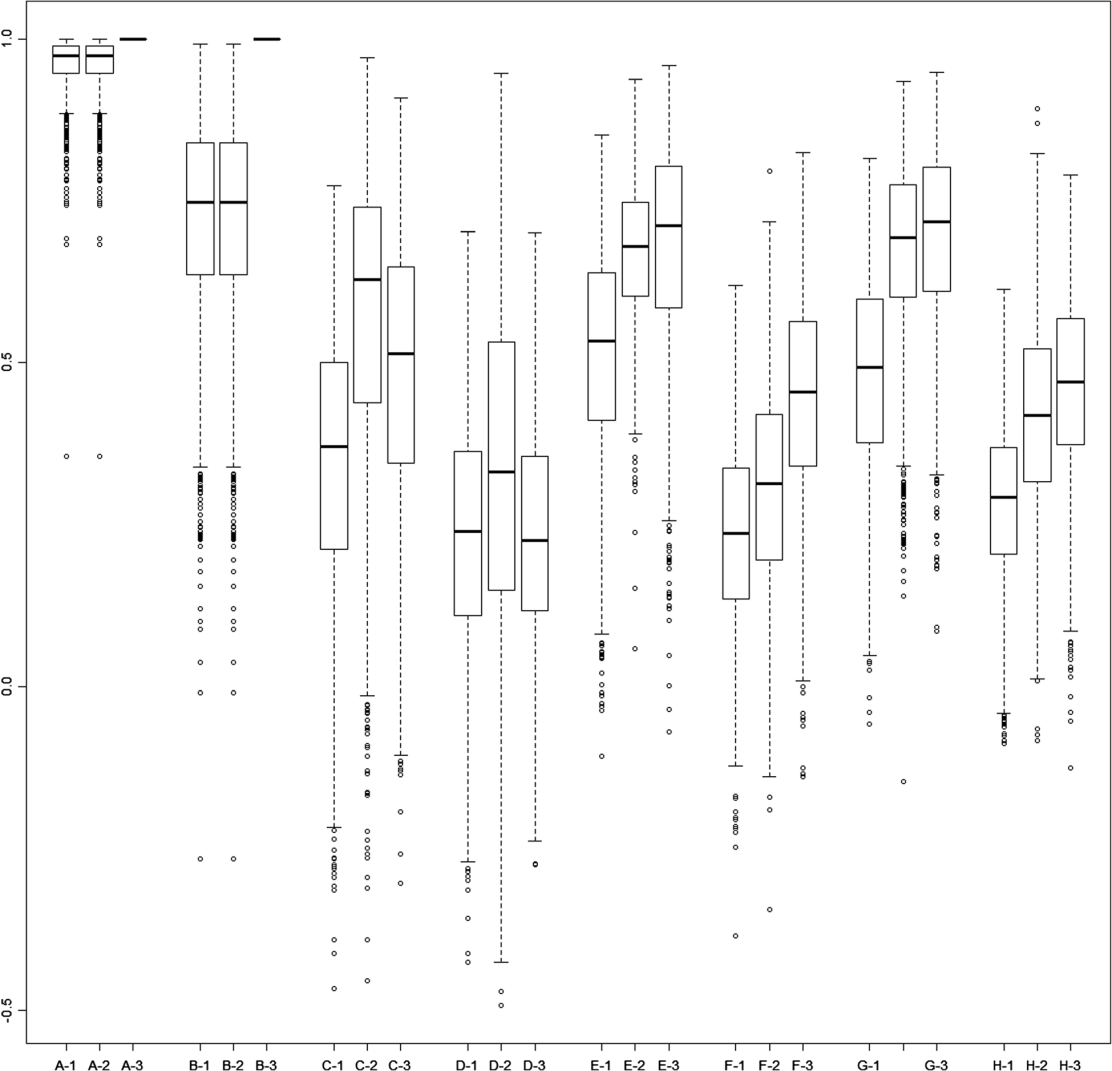
**Box plots of the correlation coefficients between risk scores of Moffitt Cancer Center (MCC) test data predicted by the models developed from the Moffitt and Vanderbilt data using re-sampling techniques based on 1,000 repetitions.** For each statistical model, three correlations are computed: 1) ρ_1_ (model transferability): consistency of risk scores developed from the VMC and MCC centers, 2) ρ_2_ (signature transferability): consistency of risk scores predicted by two centers using the same MCC signature, and 3) ρ_3_ (signature transferability): consistency of risk scores predicted from MCC data using different signatures.

Using resampling, the colon data were further evaluated to address whether or not the prediction model built from VMC could be used to predict risk scores for MCC patients. The 177 patients from MCC were randomly split into a training set of 55 patients and a test set of 122 patients. The 55 samples from VMC and the 55 training samples from MCC were separately used to develop two prediction models (*vmc* and *mcc1*) to predict risk scores for the 122 test samples. The signature genes from model *vmc* are also applied to develop model *mcc2* using the 55 training samples from MCC. In total, three sets of risk scores, resulting from the *vmc*, *mcc1*, and *mcc2* models, were estimated for the 122 test samples. The correlation between the risk scores from *vmc* and *mcc1*, denoted as ρ_1_, measures model transferability. The correlation between the risk scores from *vmc* and *mcc2* is denoted as ρ_2_, and the correlation between risk scores from *mcc1* and *mcc2* is denoted as ρ_3._ The correlation ρ_1_ has similar interpretation as the model transferability in the lung cancer (Table 6, first three rows of each center). The correlation coefficients for the lung cancer data were computed from the risk scores of the training model developed from the test center, but the correlations in the colon cancer data were computed from the risk scores of the 122 test samples predicted by the training model. ρ_2_ and ρ_3_ both measure transferability of the signature developed by *vmc* to the *mcc2* model. ρ_2_ measures the transferability of predicted risk scores between two centers using the same VMC signature and ρ_3_ measures the transferability of predicted risk scores using MCC modeled with different signatures. Re-sampling and calculations for ρ_1_, ρ_2_, and ρ_3_ were repeated 1,000 times. Boxplots of the resampled correlation coefficients for the eight models are shown in Figure 1.

ρ_1_ equals ρ_2_ and ρ_3_ is 1 for Models A and B because both models included only clinical variables and resulted in the same prediction model. In general, if two centers have the same signatures, then ρ_1_ should equal ρ_2_, and ρ_3_ should be 1. Furthermore, the samples generated within MCC should be more homogeneous than the samples between MCC and VMC; therefore, ρ_3_ is expected to be larger than ρ_1_ (Model A-H), where in Model D ρ_1_ is slightly larger than ρ_3_. In summary, Model A shows an excellent transferability between the two centers. In addition, Models D and F have poorer transferability, and these two models have the poorest prediction ability in Tables 7 and 8.

## Discussion and conclusions

Development of a risk prediction model for clinical use involves the two stages: 1) model development based on a set of signatures, and 2) model validation with a perspective clinical trial. This paper mainly considers the first stage in the model development. Model development also involves two stages: 1) model building and 2) model (analytical) validation. Model building involves fitting a Cox survival model by selected a set of relevant predictor signatures, from the present study. Model validation is to assess if the fitted model can predict relative risk of patient samples generated from the available data, which can include the present study and other studies. Since prediction model is typically developed based on a single study, model validation often refers to the assessment of predictive performance.

Two methods are commonly used to assess performance of a prediction model: the split-sample method and cross-validation method. In the split-sample procedure, the sample dataset is split into two subsets (either randomly split the entire data or a designated test dataset), a training set for model building and a test set for model validation. Cross validation involves repeatedly splitting the data into a training set and test set. The predictive performance is the “average” of the numerous training-test partitions. The split-sample procedure provides a single analysis of performance metrics, such as D_xy_ and p-values, etc. For data from a single (center) study, cross validation can be more valuable than the randomly split method. A common analysis of data from a multicenter study is often limited to evaluation of performance metrics using the split-sample method [20,21]. The multicenter study provides valuable data for further model validation. In addition to investigate the predictability of a model (Tables 2, 3, 4 and 5, 7, 8, and Additional file 1: Tables S2-S5), this paper presents statistical analysis to illustrate an assessment of cross-center reproducibility. Assessment of reproducibility across centers provides another layer of model validation.

The cross-validation method has also been applied to tune the parameters in some training methods for gene selection such as univariate selection, forward stepwise selection, principal components regression, supervised principal components regression, partial least squares regression, ridge regression and LASSO [52-54], and the approach may lead to less over fitted training models. The over fitting can result in poor prediction ability which may be caused by other reasons such as inappropriate models, and the prediction ability indices are more appropriate to assess the performance. Thus the cross-validation for tuning parameters is not applied to obtain the models in this paper, and some of these models we used are the well-established methods in [33,41].

The cross-center reproducibility is measured by correlations of the two sets of predicted scores derived from two centers, whereas the standard performance assessment considers the analysis of predicted scores from the test data. We present two terminologies to describe cross-center reproducibility: consistency and transferability. The consistency is a general term referring to an agreement between two sets of predicted scores derived from two entities (centers). Transferability refers specifically to an agreement of two sets of risk scores for a target center, one set is derived from a model developed from the target center and another set is predicted by another center. The risk scores of the target center can be the training scores derived from the fitting of entire data (lung cancer data), or they can be the predicted scores derived from fitting a partially set of data (colon cancer data). Although most cancer study does not involve more than two centers, the transferability should have more use for assessment of reproducibility between two centers in practice.

The lung cancer data consists of four medical centers. The predictability of the each of the eight models was assessed by evaluating the performance metrics between center predictions (Additional file 1: Tables S2-S5). The consistency and transferability of the predicted scores derived from two centers were further evaluated (Table 6). In this analysis, the entire data set was used in the evaluation; that is, the consistency and transferability correlations were evaluated for the entire target center. In this analysis, Model A appears to perform the best in terms of both the predictability and cross-center reproducibility. In terms of the cross-center prediction, the prediction from HLM to DFCI is the best. It could result from the high agreement for the clinical variables in the different data. A conclusion is that a good prediction model shows a high cross-center consistency. It should be emphasized that higher consistency does not necessarily imply better performance.

The colon cancer data were analyzed slightly different. The colon dataset consisted of 55 VMC patients and 177 MCC patients. We used the MCC as a target center to evaluate the prediction models built from VMC. In this analysis, 55 randomly selected MCC patients (a partial dataset) were used to develop a model to predict 122 remaining patients and compared with the model developed from 55 VMC patients. The consistency and transferability correlations were evaluated only for the 122 patients. We considered signature transferability and model transferability to assess the generalizability of prediction models. Although a major concern in the validation of a microarray-based prediction model is model transferability i.e. the usefulness of a transferred model outside of its intended use, it is also desirable that the signature developed from the internal dataset is applicable to predict future samples.

For gene expression Models C and D, the signature consistency appears to be higher than the model consistency (Figure 1). However, neither model performs well when compared to the clinical models (Tables 7 and 8). It should be emphasized that higher consistency does not necessarily imply better performance. Model G has the highest D_xy_ value, but Models A and B have better consistency in both the classifier transferability and signature transferability. In all, it appears that Model A (Cox model) performs more consistent than Model B (regression tree). Therefore, the reproducibility of cancer survival model including gene expression data across different centers or studies could be still controversial, that could be caused by the geographic and/or methodologic variations, and it should be extensively studied. Finally, the transferability of model or signature is meaningful only after the model has established its performance.

Generally, a prediction model has medical utility only if it enables clinicians to make better treatment decisions for individual patients. Establishing medical utility of a prediction model requires validation from a prospective clinical trial. Although there have been a number of publications discussing the design and analysis of clinical trials for validation of cancer prognostic and predictive models [55-58], very few clinical trials have been conducted. A major factor is due to the lack of reproducibility of the prediction model to justify conducting a prospective clinical validation trial. In this paper, we illustrate an analytical (external) validation of risk prediction modeling to assess reproducibility across studies. Based on the analyses of the two cancer datasets, we conclude that the models with clinical variables appear to perform well with high degree of consistency and transferability and inclusion of gene expression variables shows little improvement.

## Competing interests

The authors declare that they have no competing interests.

## Authors’ contributions

JJC conceived the study and wrote the manuscript. HCC developed and implemented the methodology and performed the analysis. Both of the authors read and approved the manuscript.

## Pre-publication history

The pre-publication history for this paper can be accessed here:

http://www.biomedcentral.com/1471-2288/13/25/prepub

## Supplementary Material

Additional file 1 **For patient *j*, the survival time can be represented by (T**_***j***_**, δ**_***j***_**) where T**_***j***_**event and 0: censoring), and the predictive risk score is H**_***j***_**.** The true positive rate, TPR(t,c), and the false positive rate, FPR(t,c), for some cut, c, of the risk scores are defined as TP(t,c)/(TP(t,c)+FN(t,c)) and FP(t,c)/(FP(t,c)+TN(t,c)), respectively. Table S1. The eight risk prediction models. Table S2a. Prediction ability of HLM training model for UM. Table S2b. Prediction ability of DFCI training model for UM. Table S2c. Prediction ability of MSK training model for UM. Table S3a. Prediction ability of UM training model for HLM. Table S3b. Prediction ability of DFCI training model for HLM. Table S3b. Prediction ability of MSK training model for HLM. S4a. Prediction ability of UM training model for DFCI. S4b. Prediction ability of HLM training model for DFCI. S4c. Prediction ability of MSK training model for DFCI. S5a. Prediction ability of UM training model for MSK. S5b. Prediction ability of HLM training model for MSK. S5c. Prediction ability of DFCI training model for MSK. Figure S1. ROC curves for the eight models estimated at month 36. (a) Training data UM/HLM, test data DFCI/MSK. Models A and F have better performance. (b) Training data: DFCI/MSK, test data UM/HLM. Models A and E have better performance. Figure S2a. Scatter plots of training scores and test scores for UM. Figure S2b. Scatter plots of training scores and test scores for HLM. Figure S2c. Scatter plots of training scores and test scores for DFCI. Figure S2d. Scatter plots of training scores and test scores for MSK. Figure S3. Kaplan–Meier survival curves and p-value from the log-rank test for MCC patients from each of the eight prediction models. Each patient was classified into the high- or low-risk group based on the median risk score in the training data generated from the VMC patients. Figure S4. ROC curves for patient survival using each of the eight models. Patient survival is evaluated at 25th, 50th, and 75th percentiles of follow-up time, corresponding to month 23, 42, and 68 after surgical removal of colon tumors. Click here for file
