# Supplementary material for: Assessment of reproducibility of cancer survival risk predictions across medical centers
Source: BMC Med Res Methodol. 2013 Feb 20;13:25. doi: 10.1186/1471-2288-13-25 (PMC3598915; doi:10.1186/1471-2288-13-25)

**Supplementary**

For patient *j*, the survival time can be represented by (T*_j_*, δ*_j_*) where T*_j_* is the follow-up time and δ*_j_* is the indicator for event (1:event and 0: censoring), and the predictive risk score is H*_j_*. The true positive rate, TPR(t,c), and the false positive rate, FPR(t,c), for some cut, c, of the risk scores are defined as TP(t,c)/(TP(t,c)+FN(t,c)) and FP(t,c)/(FP(t,c)+TN(t,c)), respectively, where

$TP(t,c)=\sum_{j=1}^{n} I(T_{j}\leq t, \delta_{j}=1,H_{j}\geq c)+I(T_{j}<t, \delta_{j}=0,H_{j}\geq c)(1-\prod_{t_{j}<t_{k}\leq t} (1-\frac{d_{t_{k}}}{n_{t_{k}}}))$,

$FP(t,c)=\sum_{j=1}^{n} I(T_{j}>t,H_{j}\geq c)+I(T_{j}=t,\delta_{j}=0,H_{j}\geq c)+I(T_{j}<t, \delta_{j}=0,H_{j}\geq c)\prod_{t_{j}<t_{k}\leq t} (1-\frac{d_{t_{k}}}{n_{t_{k}}})$,

$FN(t,c)=\sum_{j=1}^{n} I(T_{j}\leq t, \delta_{j}=1,H_{j}<c)+I(T_{j}<t, \delta_{j}=0,H_{j}<c)(1-\prod_{t_{j}<t_{k}\leq t} (1-\frac{d_{t_{k}}}{n_{t_{k}}}))$, and

$TN(t,c)=\sum_{j=1}^{n} I(T_{j}>t,H_{j}<c)+I(T_{j}=t,\delta_{j}=0,H_{j}<c)+I(T_{j}<t, \delta_{j}=0,H_{j}<c)\prod_{t_{j}<t_{k}\leq t} (1-\frac{d_{t_{k}}}{n_{t_{k}}})$.

Table S1. The eight risk prediction models

| Model | Predictors |
| --- | --- |
| A | clinical covariates model - Cox proportional hazards |
| B | clinical covariates model - regression tree |
| C | gene expression model - 5 principal components |
| D | gene expression model - 10 most significant genes |
| E | clinical covariates-Cox + 5 principal components (A+C) |
| F | clinical covariates-Cox + 10 most significant genes (A+D) |
| G | clinical covariates-tree + 5 components (B+C) |
| H | clinical covariates-tree + 10 most significant genes (B+D) |

Table S2a. Prediction ability of HLM training model for UM

| Model | D_xy_ | Single Group Analysis | | | High- versus Low-Risk Group Analysis | | |
| --- | --- | --- | --- | --- | --- | --- | --- |
|  |  | HR | P-value | R^2^ | HR | Cox Model P-value | Log-rank P-value |
| A | -0.283 | 1.93 | 4.50E-6 | 0.116 | 2.01 | 0.001 | 0.001 |
| B | -0.288 | 1.72 | 1.02E-4 | 0.077 | 2.25 | 5.14E-5 | 3.23E-5 |
| C | -0.181 | 1.33 | 0.017 | 0.031 | 1.62 | 0.032 | 0.031 |
| D | -0.185 | 1.16 | 0.072 | 0.017 | 1.62 | 0.02 | 0.019 |
| E | -0.276 | 1.49 | 1.44E-5 | 0.098 | 2.35 | 0.001 | 0.001 |
| F | -0.233 | 1.22 | 0.001 | 0.051 | 1.85 | 0.011 | 0.01 |
| G | -0.246 | 1.54 | 4.17E-5 | 0.083 | 2.33 | 4.6E-4 | 3.07E-4 |
| H | -0.203 | 1.24 | 0.003 | 0.043 | 2.03 | 0.001 | 0.001 |

Table S2b. Prediction ability of DFCI training model for UM

| Model | D_xy_ | Single Group Analysis | | | High- versus Low-Risk Group Analysis | | |
| --- | --- | --- | --- | --- | --- | --- | --- |
|  |  | HR | P-value | R^2^ | HR | Cox Model P-value | Log-rank P-value |
| A | -0.284 | 1.74 | 3.30E-6 | 0.122 | 2.1 | 0.001 | 4.23E-4 |
| B | -0.288 | 1.75 | 1.93E-5 | 0.094 | 2.96 | 1.10E-07 | 2.63E-08 |
| C | -0.185 | 1.2 | 0.071 | 0.018 | NA | NA | NA |
| D | -0.077 | 1.02 | 0.824 | 0 | NA | NA | NA |
| E | -0.378 | 1.52 | 1.79E-8 | 0.164 | 2.86 | 4.66E-06 | 1.76E-06 |
| F | -0.156 | 1.13 | 0.019 | 0.03 | NA | NA | NA |
| G | -0.331 | 1.57 | 2.11E-7 | 0.137 | 2 | 0.002 | 0.001 |
| H | -0.171 | 1.16 | 0.038 | 0.023 | NA | NA | NA |

Table S2c. Prediction ability of MSK training model for UM

| Model | D_xy_ | Single Group Analysis | | | High- versus Low-Risk Group Analysis | | |
| --- | --- | --- | --- | --- | --- | --- | --- |
|  |  | HR | P-value | R^2^ | HR | Cox Model P-value | Log-rank P-value |
| A | -0.272 | 1.59 | 3.49E-05 | 0.09 | 2.08 | 2.49E-4 | 1.81E-4 |
| B | -0.217 | 1.69 | 4.65E-05 | 0.073 | 1.88 | 0.001 | 0.001 |
| C | -0.173 | 1.3 | 0.003 | 0.049 | 1.37 | 0.329 | 0.327 |
| D | -0.183 | 1.22 | 0.003 | 0.048 | 1.35 | 0.216 | 0.215 |
| E | -0.284 | 1.38 | 2.72E-06 | 0.115 | 1.98 | 0.006 | 0.005 |
| F | -0.273 | 1.29 | 4.52E-07 | 0.13 | 1.77 | 0.006 | 0.005 |
| G | -0.223 | 1.37 | 2.01E-05 | 0.09 | 1.42 | 0.174 | 0.172 |
| H | -0.226 | 1.28 | 3.85E-05 | 0.088 | 1.84 | 0.046 | 0.043 |

Table S3a. Prediction ability of UM training model for HLM

| Model | D_xy_ | Single Group Analysis | | | High- versus Low-Risk Group Analysis | | |
| --- | --- | --- | --- | --- | --- | --- | --- |
|  |  | HR | P-value | R^2^ | HR | Cox Model P-value | Log-rank P-value |
| A | -0.24 | 1.91 | 0.004 | 0.097 | 1.88 | 0.023 | 0.021 |
| B | -0.183 | 1.62 | 0.007 | 0.086 | 1.58 | 0.219 | 0.215 |
| C | -0.097 | 1.44 | 0.116 | 0.032 | 1.14 | 0.726 | 0.725 |
| D | 0.054 | 0.98 | 0.876 | 0 | 1.18 | 0.56 | 0.56 |
| E | -0.265 | 1.46 | 0.001 | 0.137 | 2.28 | 0.002 | 0.002 |
| F | -0.089 | 1.16 | 0.202 | 0.021 | 1.66 | 0.1 | 0.097 |
| G | -0.25 | 1.46 | 0.004 | 0.095 | 2.6 | 0.014 | 0.012 |
| H | -0.124 | 1.17 | 0.181 | 0.023 | 1.19 | 0.537 | 0.537 |

Table S3b. Prediction ability of DFCI training model for HLM

| Model | D_xy_ | Single Group Analysis | | | High- versus Low-Risk Group Analysis | | |
| --- | --- | --- | --- | --- | --- | --- | --- |
|  |  | HR | P-value | R^2^ | HR | Cox Model P-value | Log-rank P-value |
| A | -0.235 | 1.67 | 0.001 | 0.131 | 1.7 | 0.072 | 0.069 |
| B | -0.26 | 1.92 | 3.72E-4 | 0.149 | 2.41 | 0.002 | 0.001 |
| C | -0.181 | 1.22 | 0.141 | 0.027 | NA | NA | NA |
| D | -0.051 | 1.11 | 0.168 | 0.022 | NA | NA | NA |
| E | -0.229 | 1.27 | 0.006 | 0.088 | 2.11 | 0.019 | 0.016 |
| F | -0.226 | 1.1 | 0.014 | 0.063 | NA | NA | NA |
| G | -0.307 | 1.38 | 0.001 | 0.122 | 2.33 | 0.004 | 0.003 |
| H | -0.213 | 1.13 | 0.015 | 0.061 | NA | NA | NA |

Table S3b. Prediction ability of MSK training model for HLM

| Model | D_xy_ | Single Group Analysis | | | High- versus Low-Risk Group Analysis | | |
| --- | --- | --- | --- | --- | --- | --- | --- |
|  |  | HR | P-value | R^2^ | HR | Cox Model P-value | Log-rank P-value |
| A | -0.213 | 1.34 | 0.032 | 0.056 | 2.13 | 0.004 | 0.003 |
| B | -0.181 | 1.18 | 0.339 | 0.011 | 1.97 | 0.011 | 0.01 |
| C | -0.168 | 1.15 | 0.224 | 0.019 | 1.11 | 0.788 | 0.788 |
| D | -0.069 | 1.21 | 0.055 | 0.046 | 1 | 0.993 | 0.993 |
| E | -0.237 | 1.14 | 0.059 | 0.044 | 1.28 | 0.362 | 0.361 |
| F | -0.134 | 1.16 | 0.027 | 0.061 | 1.6 | 0.093 | 0.091 |
| G | -0.188 | 1.13 | 0.191 | 0.021 | 1.53 | 0.154 | 0.151 |
| H | -0.075 | 1.23 | 0.022 | 0.064 | 1.31 | 0.387 | 0.386 |

S4a. Prediction ability of UM training model for DFCI

| Model | D_xy_ | Single Group Analysis | | | High- versus Low-Risk Group Analysis | | |
| --- | --- | --- | --- | --- | --- | --- | --- |
|  |  | HR | P-value | R^2^ | HR | Cox Model P-value | Log-rank P-value |
| A | -0.409 | 3.22 | 2.62E-5 | 0.2 | 2.7 | 0.01 | 0.008 |
| B | -0.24 | 2.13 | 0.001 | 0.113 | 0.74 | 0.434 | 0.425 |
| C | -0.373 | 1.81 | 0.007 | 0.081 | NA | NA | NA |
| D | -0.309 | 1.79 | 0.004 | 0.094 | 12.68 | 0.018 | 0.005 |
| E | -0.484 | 2.07 | 3.22E-5 | 0.206 | 3.94 | 1.95E-4 | 6.14E-5 |
| F | -0.42 | 2.1 | 9.71E-6 | 0.214 | 9.17 | 0.004 | 0.001 |
| G | -0.429 | 1.95 | 5.79E-5 | 0.173 | 16.5 | 2.16E-6 | 3.07E-10 |
| H | -0.347 | 1.79 | 7.53E-5 | 0.156 | 3.61 | 0.035 | 0.026 |

S4b. Prediction ability of HLM training model for DFCI

| Model | D_xy_ | Single Group Analysis | | | High- versus Low-Risk Group Analysis | | |
| --- | --- | --- | --- | --- | --- | --- | --- |
|  |  | HR | P-value | R^2^ | HR | Cox Model P-value | Log-rank P-value |
| A | -0.433 | 3.5 | 2.88E-5 | 0.208 | 3.32 | 4.56E-4 | 2.13E-4 |
| B | -0.463 | 2.76 | 1.84E-5 | 0.2 | 3.57 | 3.15E-4 | 1.27E-4 |
| C | -0.304 | 2.3 | 0.001 | 0.111 | 19.49 | 0.008 | 1.69E-4 |
| D | -0.305 | 1.63 | 0.002 | 0.093 | NA | NA | NA |
| E | -0.486 | 2.59 | 6.71E-6 | 0.224 | NA | NA | NA |
| F | -0.369 | 1.63 | 2.39E-4 | 0.15 | NA | NA | NA |
| G | -0.501 | 3.1 | 2.52E-7 | 0.273 | NA | NA | NA |
| H | -0.411 | 1.72 | 2.80E-5 | 0.177 | NA | NA | NA |

S4c. Prediction ability of MSK training model for DFCI

| Model | D_xy_ | Single Group Analysis | | | High- versus Low-Risk Group Analysis | | |
| --- | --- | --- | --- | --- | --- | --- | --- |
|  |  | HR | P-value | R^2^ | HR | Cox Model P-value | Log-rank P-value |
| A | -0.375 | 1.71 | 0.002 | 0.112 | 4.05 | 3.13E-4 | 9.97E-5 |
| B | -0.312 | 1.38 | 0.053 | 0.04 | 2.27 | 0.02 | 0.017 |
| C | -0.097 | 0.96 | 0.76 | 0.001 | 1.29 | 0.448 | 0.449 |
| D | -0.233 | 1.11 | 0.175 | 0.022 | NA | NA | NA |
| E | -0.159 | 1.09 | 0.417 | 0.008 | NA | NA | NA |
| F | -0.315 | 1.2 | 0.008 | 0.079 | NA | NA | 0.468 |
| G | -0.171 | 1.06 | 0.563 | 0.004 | 1.75 | 0.126 | 0.122 |
| H | -0.289 | 1.15 | 0.046 | 0.043 | 2.14 | 0.453 | 0.442 |

S5a. Prediction ability of UM training model for MSK

| Model | D_xy_ | Single Group Analysis | | | High- versus Low-Risk Group Analysis | | |
| --- | --- | --- | --- | --- | --- | --- | --- |
|  |  | HR | P-value | R^2^ | HR | Cox Model P-value | Log-rank P-value |
| A | -0.364 | 3.19 | 1.9E-4 | 0.133 | 2.24 | 0.031 | 0.026 |
| B | -0.236 | 1.64 | 0.013 | 0.058 | 1.44 | 0.332 | 0.328 |
| C | -0.177 | 1.62 | 0.078 | 0.031 | 1.95 | 0.271 | 0.261 |
| D | -0.095 | 1.37 | 0.122 | 0.024 | 1.39 | 0.396 | 0.392 |
| E | -0.294 | 1.45 | 0.024 | 0.048 | 2.16 | 0.03 | 0.025 |
| F | -0.231 | 1.56 | 0.014 | 0.063 | 3.12 | 0.032 | 0.024 |
| G | -0.374 | 1.71 | 0.002 | 0.087 | 3.87 | 0.063 | 0.045 |
| H | -0.244 | 1.55 | 0.011 | 0.067 | 2.06 | 0.108 | 0.1 |

S5b. Prediction ability of HLM training model for MSK

| Model | D_xy_ | Single Group Analysis | | | High- versus Low-Risk Group Analysis | | |
| --- | --- | --- | --- | --- | --- | --- | --- |
|  |  | HR | P-value | R^2^ | HR | Cox Model P-value | Log-rank P-value |
| A | -0.379 | 2.2 | 0.001 | 0.115 | 3.15 | 0.001 | 0.001 |
| B | -0.326 | 1.89 | 0.013 | 0.062 | 2.98 | 0.005 | 0.003 |
| C | -0.362 | 1.44 | 0.025 | 0.044 | 1.78 | 0.122 | 0.116 |
| D | -0.308 | 1.2 | 0.126 | 0.021 | 1.1 | 0.876 | 0.879 |
| E | -0.338 | 1.49 | 0.003 | 0.082 | 2.09 | 0.058 | 0.052 |
| F | -0.211 | 1.14 | 0.189 | 0.017 | 0.7 | 0.633 | 0.629 |
| G | -0.352 | 1.49 | 0.009 | 0.062 | 2.04 | 0.078 | 0.071 |
| H | -0.307 | 1.23 | 0.068 | 0.031 | 0.89 | 0.844 | 0.842 |

S5c. Prediction ability of DFCI training model for MSK

| Model | D_xy_ | Single Group Analysis | | | High- versus Low-Risk Group Analysis | | |
| --- | --- | --- | --- | --- | --- | --- | --- |
|  |  | HR | P-value | R^2^ | HR | Cox Model P-value | Log-rank P-value |
| A | -0.296 | 1.77 | 0.008 | 0.074 | 1.93 | 0.102 | 0.094 |
| B | -0.315 | 1.81 | 0.008 | 0.068 | 3.58 | 1.53E-4 | 5.56E-5 |
| C | -0.22 | 1.22 | 0.296 | 0.01 | NA | NA | NA |
| D | 0.048 | 0.81 | 0.183 | 0.02 | NA | NA | NA |
| E | -0.144 | 1.13 | 0.338 | 0.009 | 0.93 | 0.844 | 0.846 |
| F | -0.03 | 0.96 | 0.661 | 0.002 | NA | NA | NA |
| G | -0.302 | 1.37 | 0.015 | 0.055 | 3.02 | 0.001 | 0.001 |
| H | -0.131 | 1.02 | 0.851 | 0 | NA | NA | NA |

Figure S1. ROC curves for the eight models estimated at month 36. (a) Training data UM/HLM, test data DFCI/MSK. Models A and F have better performance. (b) Training data: DFCI/MSK, test data UM/HLM. Models A and E have better performance.

.


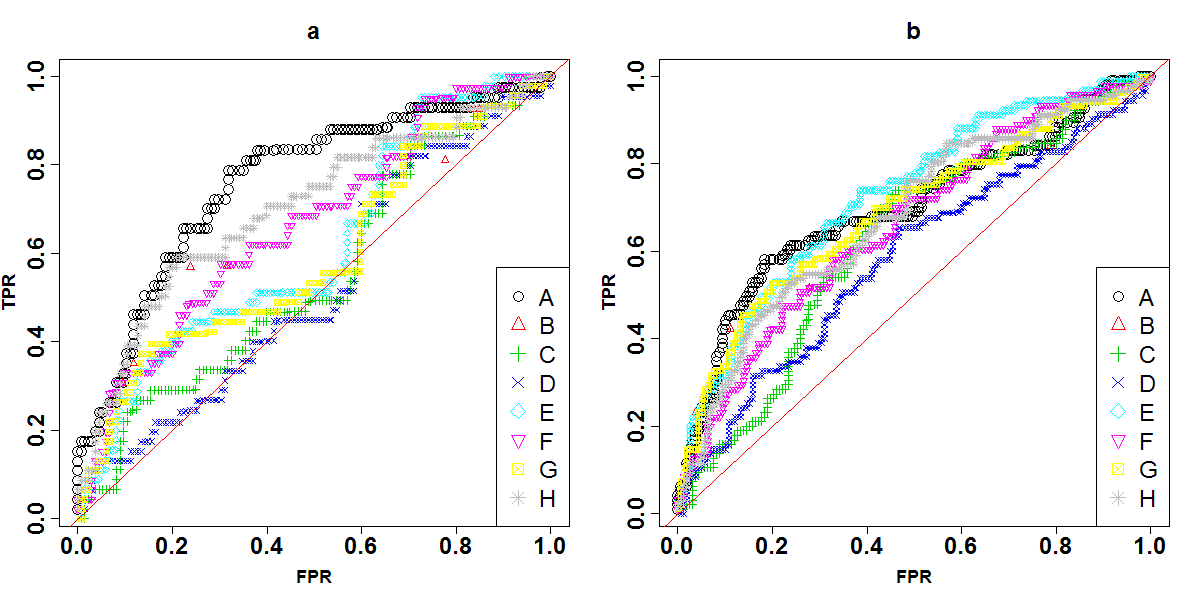


Figure S2a. Scatter plots of training scores and test scores for UM


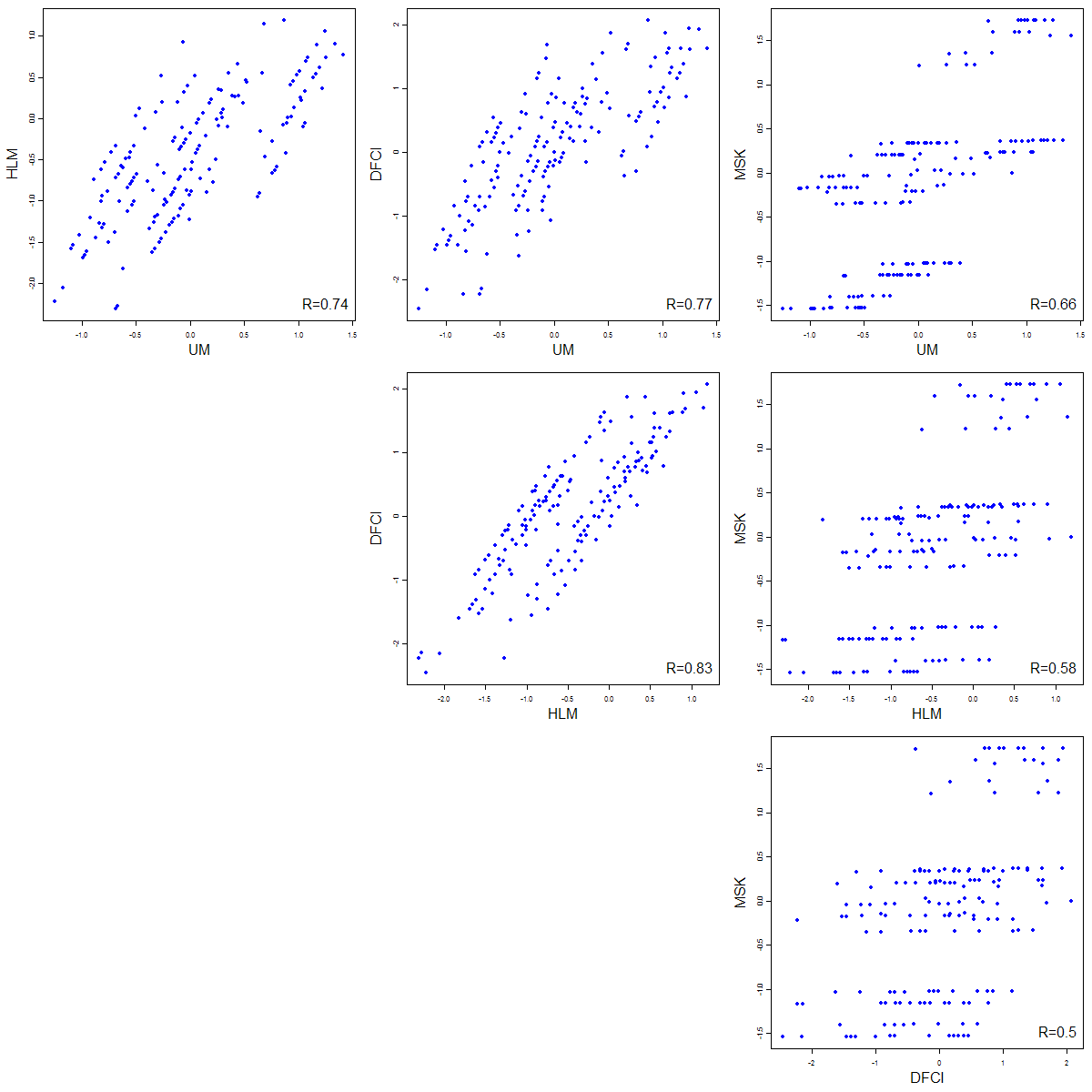


Figure S2b. Scatter plots of training scores and test scores for HLM


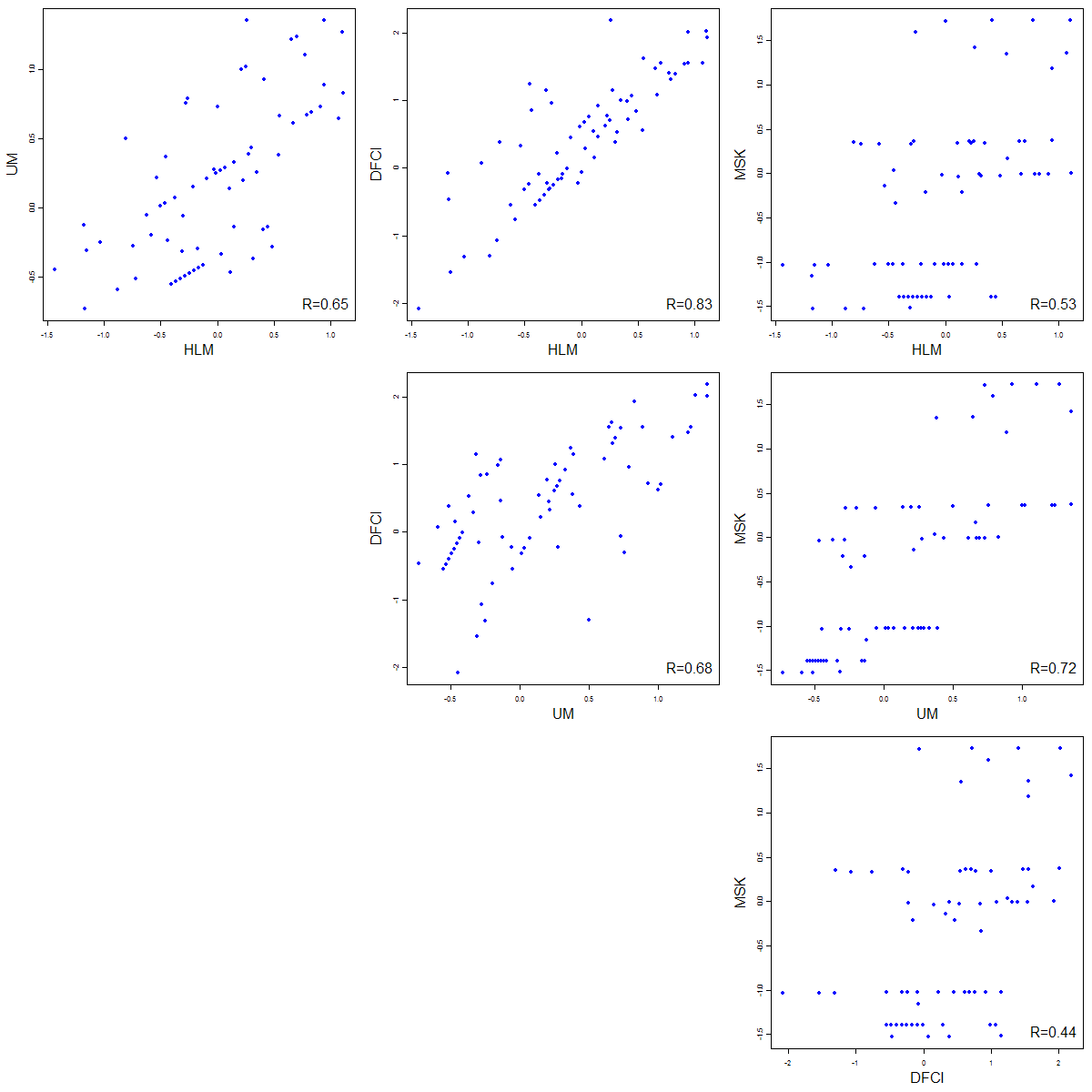


Fig S2c. Scatter plots of training scores and test scores for DFCI


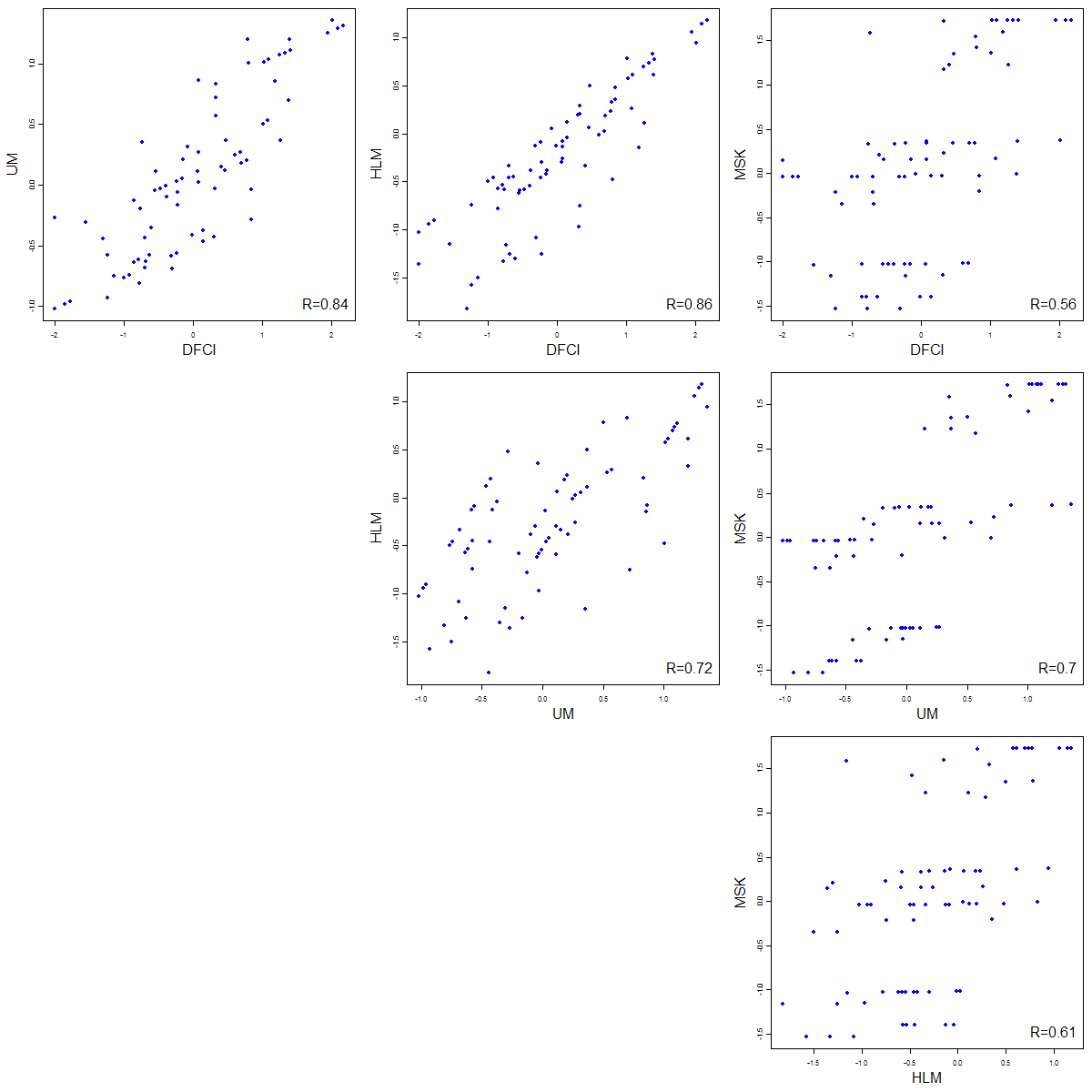


Fig S2d. Scatter plots of training scores and test scores for MSK


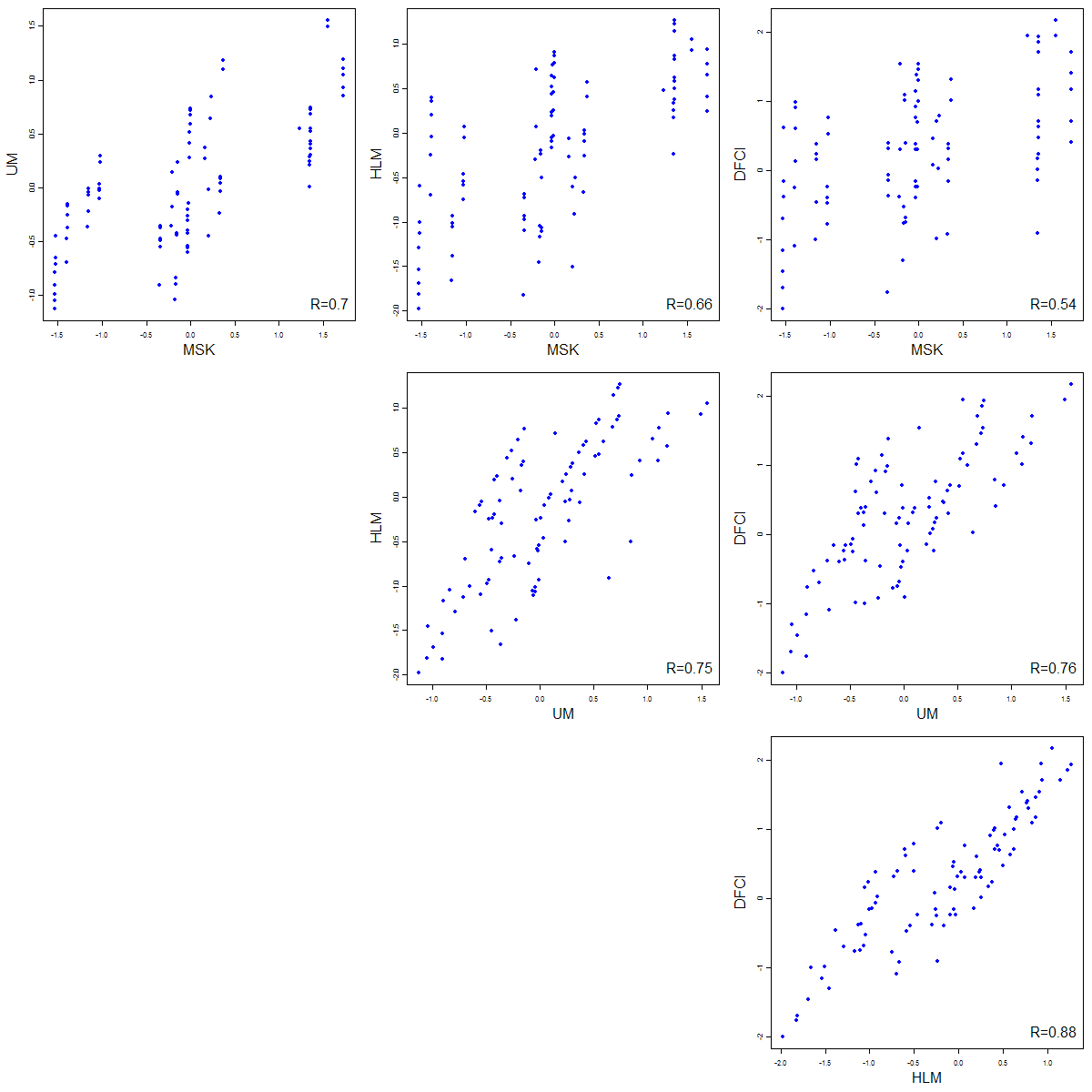


Figure S3. Kaplan–Meier survival curves and p-value from the log-rank test for MCC patients from each of the eight prediction models. Each patient was classified into the high- or low-risk group based on the median risk score in the training data generated from the VMC patients.


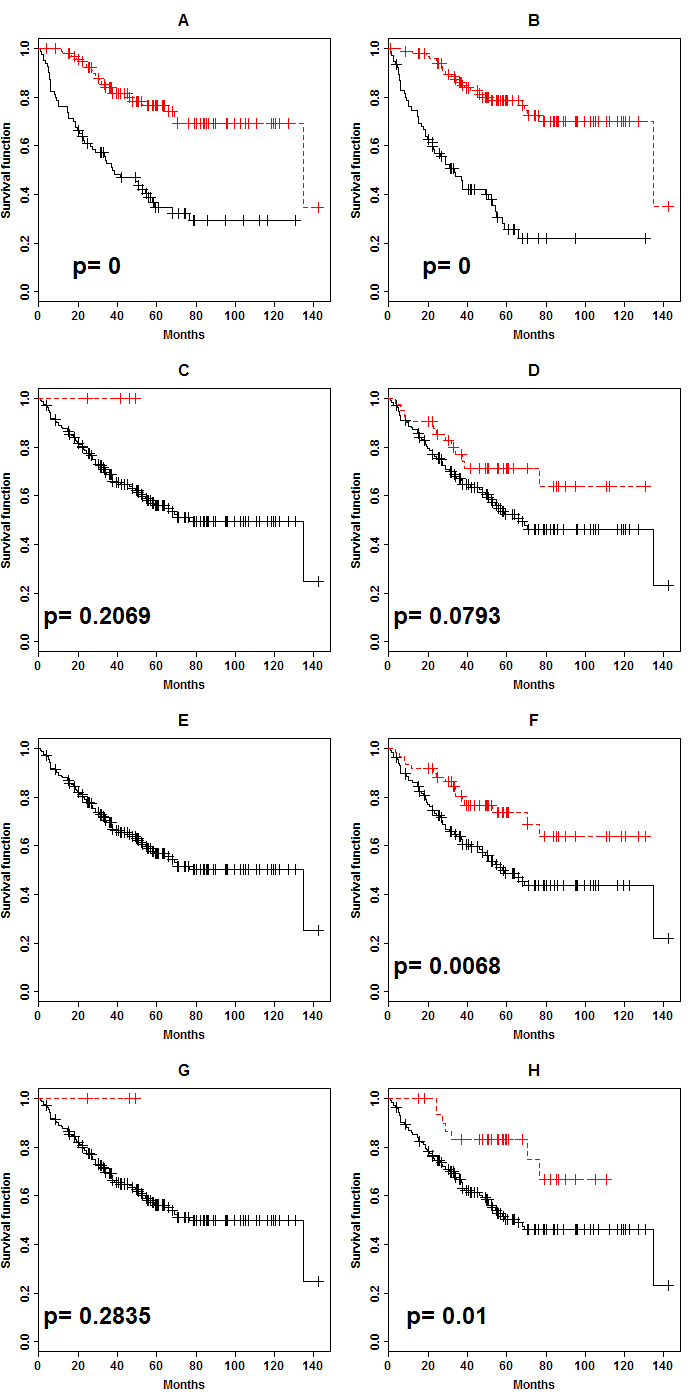


Figure S4. ROC curves for patient survival using each of the eight models. Patient survival is evaluated at 25^th^, 50^th^, and 75^th^ percentiles of follow-up time, corresponding to month 23, 42, and 68 after surgical removal of colon tumors.


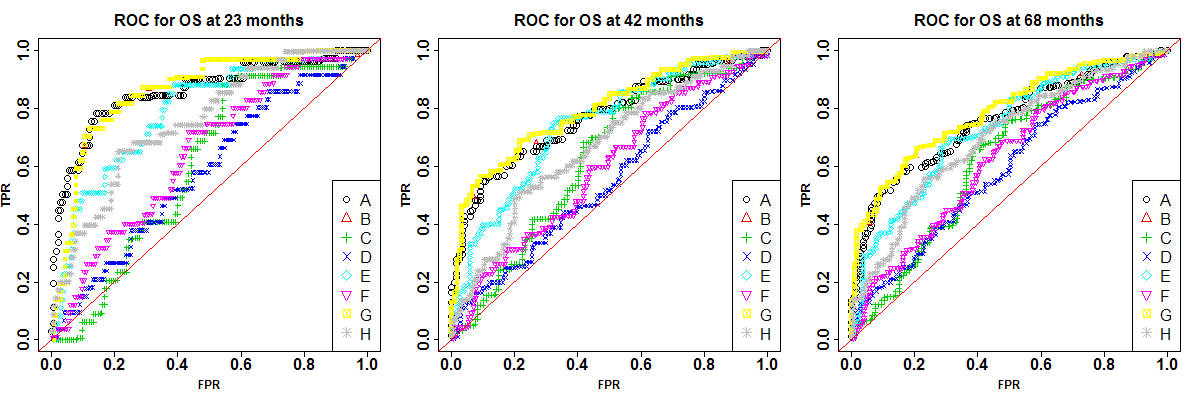

Supplement: Additional file 1 — For patient j, the survival time can be represented by (Tj, δj) where Tjevent and 0: censoring), and the predictive risk score is Hj. The true positive rate, TPR(t,c), and the false positive rate, FPR(t,c), for some cut, c, of the risk scores are defined as TP(t,c)/(TP(t,c)+FN(t,c)) and FP(t,c)/(FP(t,c)+TN(t,c)), respectively. Table S1. The eight risk prediction models. Table S2a. Prediction ability of HLM training model for UM. Table S2b. Prediction ability of DFCI training model for UM. Table S2c. Prediction ability of MSK training model for UM. Table S3a. Prediction ability of UM training model for HLM. Table S3b. Prediction ability of DFCI training model for HLM. Table S3b. Prediction ability of MSK training model for HLM. S4a. Prediction ability of UM training model for DFCI. S4b. Prediction ability of HLM training model for DFCI. S4c. Prediction ability of MSK training model for DFCI. S5a. Prediction ability of UM training model for MSK. S5b. Prediction ability of HLM training model for MSK. S5c. Prediction ability of DFCI training model for MSK. Figure S1. ROC curves for the eight models estimated at month 36. (a) Training data UM/HLM, test data DFCI/MSK. Models A and F have better performance. (b) Training data: DFCI/MSK, test data UM/HLM. Models A and E have better performance. Figure S2a. Scatter plots of training scores and test scores for UM. Figure S2b. Scatter plots of training scores and test scores for HLM. Figure S2c. Scatter plots of training scores and test scores for DFCI. Figure S2d. Scatter plots of training scores and test scores for MSK. Figure S3. Kaplan–Meier survival curves and p-value from the log-rank test for MCC patients from each of the eight prediction models. Each patient was classified into the high- or low-risk group based on the median risk score in the training data generated from the VMC patients. Figure S4. ROC curves for patient survival using each of the eight models. Patient survival is evaluated at 25th, 50th, and 75th per [file 1471-2288-13-25-S1.doc]
